# Involvement of *hpap2* and *dgkA* Genes in Colistin Resistance Mediated by *mcr* Determinants

**DOI:** 10.3390/antibiotics9090531

**Published:** 2020-08-22

**Authors:** Alejandro Gallardo, María Ugarte-Ruiz, Marta Hernández, Pedro Miguela-Villoldo, David Rodríguez-Lázaro, Lucas Domínguez, Alberto Quesada

**Affiliations:** 1 Departamento de Bioquímica, Facultad de Veterinaria, Universidad de Extremadura, Av. de la Universidad s/n, 10003 Cáceres, Spain; alexgsoler@unex.es; 2 VISAVET Health Surveillance Centre, Universidad Complutense de Madrid, 28040 Madrid, Spain; mugarter@ucm.es (M.U.-R.); pedromig@ucm.es (P.M.-V.); lucasdo@visavet.ucm.es (L.D.); 3 Laboratorio de Biología Molecular y Microbiología, Instituto Tecnológico Agrario de Castilla y León, 47071 Valladolid, Spain; ita-HerPerMa@itacyl.es; 4 Unidad de Microbiología, Departamento de Biotecnología y Ciencia de los Alimentos, Universidad de Burgos, 09001 Burgos, Spain; drlazaro@ubu.es; 5 INBIO G + C, Universidad de Extremadura, 10003 Cáceres, Spain

**Keywords:** colistin (polymyxin) resistance, *mcr-1*, *mcr-3*, *pap2*, *dagK*, lipid A (LPS), PmrAB, phosphoethanolamine (PEtN), undecaprenyl pyrophosphate (UPP), bacitracin resistance

## Abstract

Plasmid-mediated colistin resistance (*mcr*) determinants are challenging the efficacy of polymyxins against Gram-negative pathogens. Among 10 *mcr* genes described so far, the major determinants *mcr-1* and *mcr-3* are found closely linked to *hpap2* or *dgkA* genes, encoding a hypothetical phosphatidic acid phosphatase of type 2 (PAP2) and a diacylglycerol kinase, respectively, whose functions are still unknown. In this study, *mcr-1*, *mcr-1–hpap2*, *mcr-3*, and *mcr-3–dgkA* were expressed in *Escherichia coli*, and recombinant strains were analyzed to detect antimicrobial susceptibility and changes in the expression of genes involved in phospholipid metabolism. The *mcr-1* or *mcr-3* single genes were enough to drive growth on colistin selective media, although co-expression of linked genes conferred maximal antibiotic resistance. Expression of *mcr* determinants downregulated endogenous genes involved in lipopolysaccharide (LPS) modification or phospholipid recycling, although to different extents of repression: strong for *arnB*, *ybjG*, and *pmrR*; medium for *eptA*, *lpxT*, and *dgkA*; small for *bacA* and *pgpB.* Four of these genes (*bacA*, *lpxT*, *pgpB*, and *ybjG*) encode undecaprenyl pyrophosphate (UPP) phosphatases. In these conditions, cells presented resistance against bacitracin, an antibiotic that sequesters UPP from PAP2 enzymes. The *hpap2* and *dgkA* genes might play a role in colistin resistance by compensating for phospholipid metabolism functions altered during LPS modification by colistin resistance determinants.

## 1. Introduction

In the narrow frame of only four years, the wide spread of *Enterobacteriaceae* spp. resistant to polymyxins became a world-scale challenge to the efficacy of these compounds as last-resort antimicrobials [1]. Polymyxin B and E (colistin) are closely related cyclic peptides, rich in positively charged residues that interact with the lipopolysaccharide (LPS) embedded in the outer leaf of the external membrane of Gram-negative bacteria. The strength of ionic bonds between LPS and cationic molecules like polymyxins is strictly regulated by two-component sensor systems (TCSS) and effector enzymes that modify the LPS decoration status [2]. The *eptApmrAB* operon of *Enterobacteriaceae* spp. encodes a TCSS where PmrB senses high concentration of Fe^3+^ or Al^3+^ or mildly acidic pH and PmrA upregulates transcription of its own operon, including *eptA* (also known as *pmrC*) for a phosphoethanolamine (PEtN) transferase to the (1′-phosphate) lipid A. Additionally, *pmrA* also regulates expression from the *arnBCADTEF* operon (*pmrHFIJKLM*) and *ugd* (*pmrE*) gene, encoding enzymes for synthesis and transfer of L-4-aminoarabinose (L-Ara4N) to the 4′-phosphate of lipid A, which reduce the negative charge of the LPS, in addition to EptA. Less interaction with external cations such as iron in turn decreases PmrAB activation, a negative feedback that balances excess LPS decoration [3]. This regulatory circuit could be lost by mutations in critical residues that produce the constitutive activation of PmrA or PmrB and, consequently, colistin resistance. Although well known for a long time, the prevalence of colistin resistance produced by chromosomal mutations among natural strains remained anecdotic, making believe acceptable their massive use as prophylactic agents for husbandry and poultry while also being utilized as the last therapeutic option for critical infections in humans [4]. The time-off for polymyxins ended with the detection of plasmid-mediated colistin-resistance determinants (*mcr*), carried by different plasmids that in many cases are transferred by conjugation with high efficiency and belonging to the same gene family but far related to *eptA* [1].

EptA/MCR enzymes are integral membrane proteins with the active site oriented to the bacterial periplasm [1]. In the outer leaf of the plasmatic membrane, their activity transfers PEtN from phosphatidylethanolamine to 1′-phosphate lipid A and produces diacylglycerol (DAG), a membrane-disrupting lipid. This explains why the *eptA* gene from *Enterobacteriaceae* spp. is closely co-regulated with *dgkA*, encoding the diacylglycerol kinase (DAGK) that recycles DAG [5]. On the other hand, the reaction catalyzed by EptA/MCR competes for 1′-phoshate lipid A with the LpxT enzyme, which phosphorylates it to 1′-bis-phosphate, increasing the negative charge of LPS and preventing PEtN addition, which in turn decreases resistance to polymyxins [6]. Thus, EptA and LpxT seem to have antagonistic functions and, accordingly, whereas LpxT is post-translationally inactivated by the *pmrR* gene product, EptA and PmrR activities are upregulated by PmrAB [3]. LpxT belongs to the PAP2 (phosphatidic acid phosphatase of type 2) protein family that spans two other members in *Escherichia coli*, YbjG and PgpB [7]. Together with BacA, another protein that has different evolutionary relationships, the four enzymes were shown as phosphatases of undecaprenyl pyrophosphate (UPP), the biosynthesis platform where precursors are assembled for extracellular structures like peptidoglycan and L-Ara4N decoration of LPS, among others. In Gram-negative bacteria, UPP requires dephosphorylation to re-enter biosynthetic pathways, with LpxT providing this phosphate for 1′-bis-phospate lipid A and YbjG, whose transcription is also upregulated by PmrAB, balancing the decrease in LpxT activity in conditions of PmrAB activation [7].

Genes *hpap2* and *dgkA* encoding hypothetical PAP2 and DAGK enzymes, respectively, are found frequently clustered to *mcr-1* and *mcr-3* determinants [8]. The possible role of *hpap2* and *dgkA* genes is controversial since, although the expression of single coding sequences from several *mcr* genes is enough to confer colistin resistance [1,9], this was severely impaired by deletion of *hpap2* from its gene-cassette downstream *mcr-1* [10].

Numerous reports described the prevalence of *mcr* genes in different hosts and environments, their plasmidic organization and mobilization, and the three-dimensional (3D) structure of the proteins encoded, where critical residues for PEtN recognizing and transfer were identified [1]. However, little is known about the interactions of *mcr* gene products with the endogenous systems involved in LPS decoration and/or phospholipid metabolism. This work addresses the impact of *mcr*-linked genes on colistin resistance, connecting their functions with the regulatory scenario that occurs after the expression of *mcr* determinants.

## 2. Results

### 2.1. Genes Closely Linked to Colistin Resistance Determinants Are Widely Conserved among Members of the mcr/Epta Family

**Figure 1:**
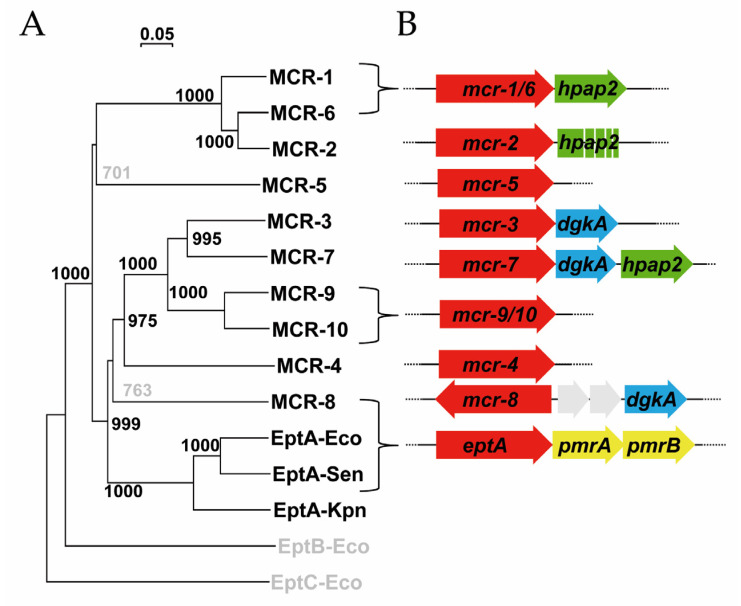
Phylogenetic relationships of plasmid-mediated colistin resistance (MCR/PmrC) proteins and clustering of their coding sequences with linked genes. (**A**) Protein sequence alignment and phylogenetic tree were generated using Clustal X 2.0 and NJPlot 2.3 (neighbor-joining algorithm and correction for multiple substitutions). Bootstrap values are indicated and assumed to support the branching of a clade when they are higher than 70% [12], whereas the scale bar represents the genetic distance (0.2 = 20%). Sequences represented are as follows: MCR-1, A0A0R6L508.1; MCR-2, WP_065419574; MCR-3, AUS91608.1; MCR-4, WP_099156046.1; MCR-5, WP_137521778.1; MCR-6, WP_099982813.1; MCR-7, WP_104009851.1; MCR-8, WP_114699275.1; MCR-9, YP_001965799.1; MCR-10, WP_023332837; EptA-Eco, AIL14661; EptA-Sen, NP_463158; EptA-Kpn, SBG93762; EptB-Eco, AIL14079.1; EptC-Eco (CptA-Eco), AIL18442.1. EptB-Eco and EptC-Eco are far-related phosphoethanolamine (PEtN) transferases to outer regions of lipopolysaccharide (LPS), included as outlier sequences. All sequences shown are from *Escherichia coli* except for MCR-6 (*Moraxella* sp.), MCR-7, MCR-8, MCR-9, and EptA-Kpn (*Klebsiellapneumoniae*) and EptA-Sen (*Salmonella enterica*). (**B**) Genetic organizations shown were deduced from the environments of *mcr*/*eptA*(*pmrC*) genes encoding the above-mentioned proteins.

Plasmid-mediated colistin resistance determinants *mcr-1* to *mcr-10* that were detected so far in *Enterobacteriaceae* spp. are orthologs of *eptA,* the chromosomal gene encoding a PEtN transferase to the 1′-phosphate of lipid A [1,11]. The phylogenetic relationships among *mcr*- and *eptA*-encoded proteins were re-analyzed in this work by bootstrapping their similarities considering the accepted cut-off value of 70% for consistent clustering [12], which suggests two main clades—MCR-1/2/6 and MCR-3/4/7/9/10 plus EptA—with MCR-5 and MCR-8 more imprecisely positioned (Figure 1A), a tree mostly consistent with previously published data [11]. Similarity relationships among *mcr* genes reached closely linked sequences, in most cases preserved inside every clade. Thus, *hpap2* is the coding sequence for a hypothetical PAP2 protein found closely linked to *mcr-1/2/6* determinants, whereas *dgkA* encodes a DAGK downstream *mcr-3/7.* Searching the nr/nt database of NCBI using BLASTn (https://blast.ncbi.nlm.nih.gov/Blast.cgi, on June 23, 2020), the *mcr-1–hpap2* gene-cassette sequence was found in 562 out of 578 complete genomes and/or plasmid sequences from *Enterobacteriaceae* spp. carrying *mcr-1*, whereas *mcr-3–dgkA* was located in the 58 complete sequences containing the *mcr-3* element (excluding *Aeromonas*, considered a reservoir of *mcr-3* with multiple sequences per genome) [13]. The *mcr*-*8* gene, which is positioned with low significance within this last clade, also presented an associated *dgkA* although located further downstream; this also applied to *mcr-7* from the *mcr-3* clade, which presents both *hpap2* and *dgkA* sequences (Figure 1B).

Interestingly, a recent study evidenced that, among 64628 genomes deposited in NCBI databases and screened for *mcr* sequences, three of the less commonly found genes lack *hpap2-* or *dgkA*-linked sequences, like *mcr-4* and *mcr-5*, or are clustered to a truncated *hpap2* coding sequence in the even more rarely found *mcr-2* gene [13]. Furthermore, *mcr-9* and *mcr-10*, the two last and most closely related *mcr* genes identified so far, lacked both *hpap2* or *dgkA* linked sequences and, although globally spread, had a weak role in colistin resistance [11,13,14]. Thus, *mcr-1* and *mcr-3* are the colistin resistance determinants more frequently mobilized, with plasmids carrying *mcr-1–hpap2* or *mcr-3–dgkA* widely distributed among different species. The biological success of these *mcr* gene clusters suggests that expressed enzymes from *hpap2* and *dgkA* genes could play a role connecting phospholipid metabolism and antimicrobial susceptibility to polymyxins.

### 2.2. Functionality of Plasmids Carrying mcr Genetic Elements

**Figure 2:**
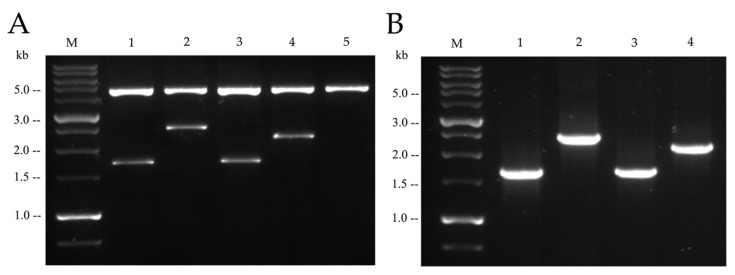
Cloning and expression of *mcr-1–hpap2* and *mcr-3–dgkA* gene constructs in pBAD24. (**A**) Agarose gel electrophoresis of pBAD24 recombinant plasmids used in this work carrying the following gene constructs: M, DNA ladder (1 kb); 1, *mcr-1*; 2, *mcr-1–hpap2*; 3, *mcr-3*; 4, *mcr-3–dgkA*; 5, empty pBAD24. DNA preparations were purified with enzymes utilized for cloning, *Eco*RI plus *Sal*I. (**B**) Agarose gel electrophoresis of rtPCR, where reverse transcription was performed on RNA purified from the four recombinant strains after arabinose induction, corresponding to one of the biological replicates from every strain utilized in experiment shown in Figure 5; PCR amplification utilized the same primers used for cloning the four *mcr* gene constructs (Table 1).

**Figure 3:**
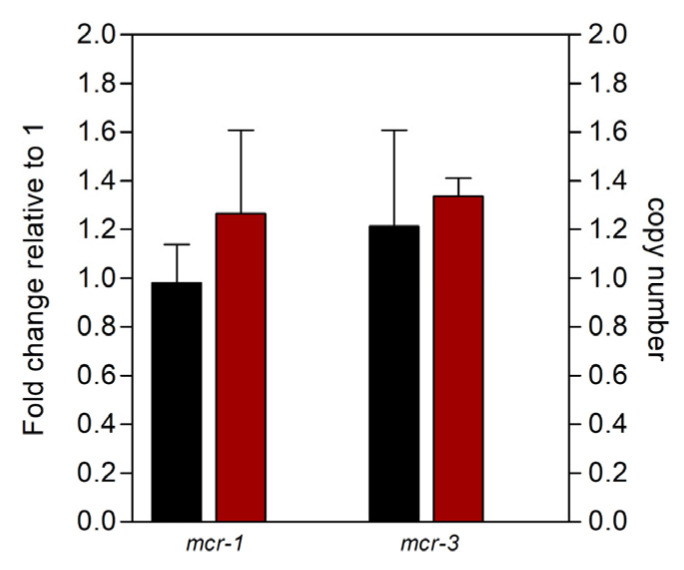
Functionality DNA constructs for *mcr* expression. Concentration (copy number) and messenger RNA (mRNA) expression from the different *mcr* gene constructs were determined by qPCR with primers mcr1q, mcr3q, and recAq (Table 1) on DNA or complementary DNA (cDNA) (see above) from the four recombinant clones. Firstly, *mcr* DNA concentration (black bars) and *mcr* gene expression (white bars) from all strains were relativized to *recA* DNA or *recA* mRNA, respectively; secondly, every two-gene construct was normalized with respect to its single *mcr*-gene reference.

The *mcr-1, mcr-1–hpap2, mcr-3*, or *mcr-3–dgkA* sequence elements were amplified and cloned in pBAD24 (see details in Section 4; Table 1 and Figure 2A), a vector used previously to express, under control of the PBAD promoter, the transcriptional regulator AraC and l-arabinose induction, and the coding sequences of *mcr-1* and *mcr-3*, among others [1]. Replication of plasmids carrying the two-gene constructs was as efficient as their relatives with single sequences (Figure 3), whereas *E. coli* strains carrying the different recombinant plasmids and induced by l-arabinose transcribed continuous messenger RNAs (mRNAs) from single- or two-gene constructs, indicating the co-transcription along *mcr-1–hpap2* or *mcr-3–dgkA* genetic elements (Figure 2B).

Furthermore, plasmids carrying two-gene constructs performed similarly for controlling expression of recombinant DNA since mRNA abundance from *mcr* genes did not change following the co-transcription with downstream sequences (Figure 3). Thus, *hpap2* and *dgkA* coding sequences are, like upstream *mcr* genes, efficiently transcribed from the PBAD promoter.

### 2.3. Expression of hpap2 or dgkA Is Not Essential, Although It Slightly Increases Resistance to Colistin in E. coli

**Figure 4:**
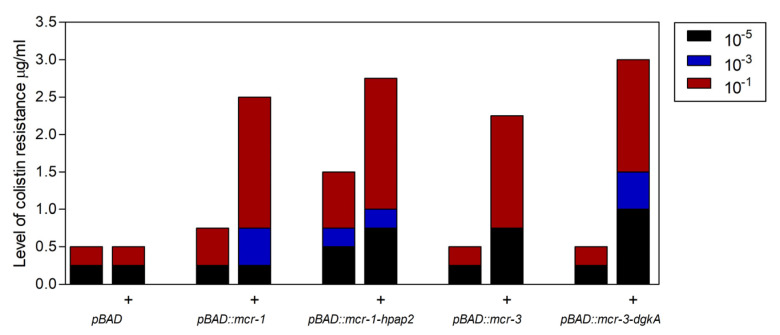
Level of colistin resistance from *mcr-1–hpap2* and *mcr-3–dgkA* gene constructs. XL1-BlueMRF’ cells carrying the different recombinant plasmids were grown, induced (+) or not with 0.2% arabinose, inoculated in three serial dilutions, and spotted on Mueller–Hinton agar plates containing 0.02% arabinose supplemented with colistin at various levels, following the protocol described in the Section 4. *pBAD* corresponds to XL1-Blue MRF’ cells carrying intact pBAD vector.

After induction with arabinose, *E. coli* strains that carry pBAD24 vectors expressing similar amounts of one or two gene constructs were inoculated in colistin media to detect antimicrobial resistance phenotypes conferred by functional expression of *mcr* and closely linked genes (Figure 4). Since a negative effect on cell viability was found after induction of *mcr* gene expression from recombinant plasmids, l-arabinose concentration in colistin-containing media was set at 0.02%, a concentration found to yield the best balance between transcription and toxicity [15]. In a gradient of colistin concentration between 0 and 3 mg/L and intervals every 0.25 mg/L, cells carrying *mcr-3–dgkA* or *mcr-1–hpap2* reached maximal colistin resistance (2.75 and 2.5 mg/L, respectively), slightly higher than that of their relative single-gene constructs *mcr-3* or *mcr-1* (2.0 and 2.25, respectively).

Moreover, pre-induction with l-arabinose was required for strains carrying *mcr-3* elements to reach resistance to colistin, whereas cultures expressing *mcr-1* were only faintly more resistant to colistin than control cells (0.5 and 0.25 mg/L, respectively), although resistance was increased up to 1.5 mg/L for the *mcr-1–hpap2* gene construct (Figure 4). On the other hand, a pBAD24 construct carrying the single coding sequence of *hpap2* did not increase colistin resistance in the same conditions assayed for *mcr*-carrying strains (not shown).

### 2.4. Expression of mcr Downregulates Genes for Phospholipid Metabolism Enzymes

**Figure 5:**
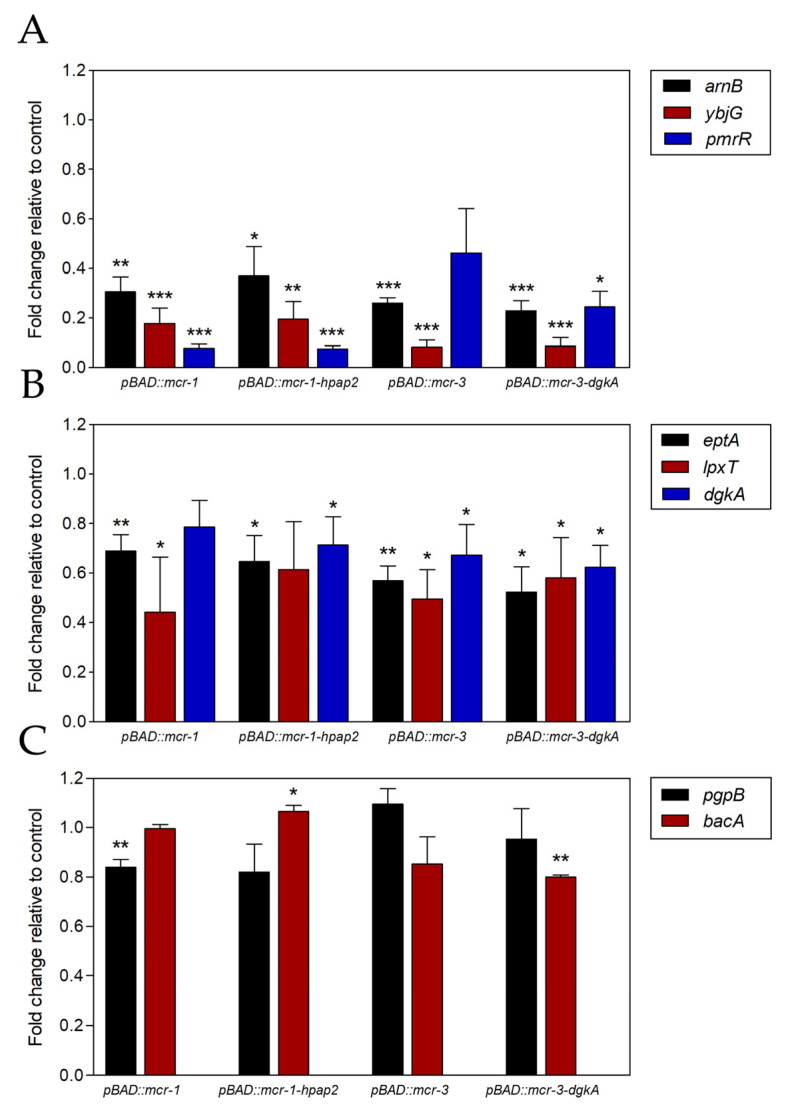
mRNA accumulation for phospholipid metabolism enzymes after expression of *mcr-1–hpap2* and *mcr-3–dgkA* gene constructs. Gene expression was quantified by qPCR with primers and conditions mentioned in Table 1, whereas cell growth and induction, RNA extraction, cDNA synthesis, amplification, and quantification are explained in detail in Section 4. The significance of differences between every condition and control cells carrying empty pBAD24 vector is indicated by * *p*  <  0.1, ** *p* < 0.05, and *** *p* < 0.01 (Student’s *t*-test). Error bars are standard deviations based on three fully independent biological replicates. Expression of genes is separately shown depending on maximal reduction achieved: (**A**) 5.0-fold; (**B**) 1.7-fold; (**C**) 1.25-fold.

Genes controlled by PmrAB, the TCSS that signals Fe^3+^ binding to cell surface [3], were found negatively regulated by the expression of *mcr* genes (Figure 5), including those for reducing the negative charge of lipid A (*eptA, arnB*, and *pmrR*), for the recycling of DAG (*dgkA*)*,* and for increasing the negative charge of lipid A (*lpxT*) [16]. Since this is related to hPAP2, whose coding sequence co-expressed with *mcr-1* improved colistin resistance (see above), the expression analysis was extended to the four genes from the *E. coli* genome that are involved in UPP dephosphorylation [7], which were also found downregulated, although to a different extent. The strongest effect was detected for *pmrR*, *YbjG*, and *arnB* genes, reaching 80% reduction (fivefold) of transcript accumulation in at least one analyzed condition (Figure 5A).

A weaker effect was observed for *dgkA*, *eptA*, and *lpxT* gene expression, whose reduction was limited to 40% (1.7-fold), whereas *bacA* and *pgpB* genes shared a minimal although still significant downregulation of 20% (1.25-fold). The composition of *mcr* gene constructs conditioned this signaling; *mcr-3* expression decreased more strongly than *mcr-1* the accumulation of mRNA from *ybjG*, *arnB*, *eptA*, and *bacA* genes, whereas the *mcr-1* effect was higher on *pmrR* and *pgpB* gene expression. In general, little or no differences were found upon comparing the co-expression of *mcr-1/-3* genes alone or linked to *hpap2* or *dgkA*, respectively. Thus, with subtle differences between *mcr-1* and *mcr-3* genes, their expression had a profound impact on the regulation of enzymes for phospholipid metabolism, decreasing the transcription of enzymes for decoration of lipid A with L-Ara4N and PEtN and for UPP dephosphorylation.

### 2.5. Expression of mcr Genes, But Not hpap2, Confers Bacitracin Resistance

**Figure 6:**
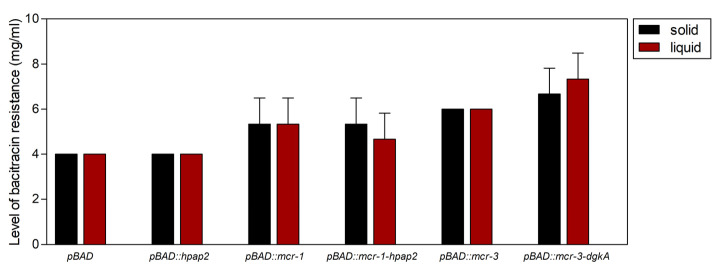
Level of bacitracin resistance from *mcr-1–hpap2* and *mcr-3–dgkA* gene constructs. XL1-Blue MRF’ cells carrying the different recombinant plasmids were grown, induced with 0.2% arabinose, and spotted on Mueller–Hinton agar plates or liquid medium containing 0.02% arabinose supplemented with bacitracin at various levels, following the protocol described in Section 4. pBAD corresponds to XL1-Blue MRF’ cells carrying intact pBAD vector.

Resistance to bacitracin, an antimicrobial peptide that binds to UPP and sequesters it from PAP2 enzymes [7], was previously shown to occur, although to a limited extent, in *E. coli* strains expressing *mcr-1* whose resistance increased from 1 to 2 mg/mL [17]. Accordingly, expression of *mcr-1* or *mcr-3* from pBAD24 vector conferred a modest increase in bacitracin resistance, from 2 up to 4 mg/mL, independently of the linked gene *hpap2*, which cloning in the same vector designed for optimal expression and translation of its coding sequence did not show any increase in bacitracin resistance (Figure 6). Furthermore, co-expression of *mcr*-3 and *dgkA* further increased bacitracin resistance to 6 mg/mL.

## 3. Discussion

The *mcr-1* and *mcr-3* genes can be traced back, with their linked sequences *hpap2* and *dgkA*, to far-related organisms like *Moraxella* and *Aeromonas*, respectively [1]. The conservation of these gene clusters during evolution (Figure 1), assuming that natural selection tends to eliminate superfluous and costly characters, suggests the existence of still unknown functional connections, the discovery of which could be the key to designing new strategies against antimicrobial resistance.

**Figure 7:**
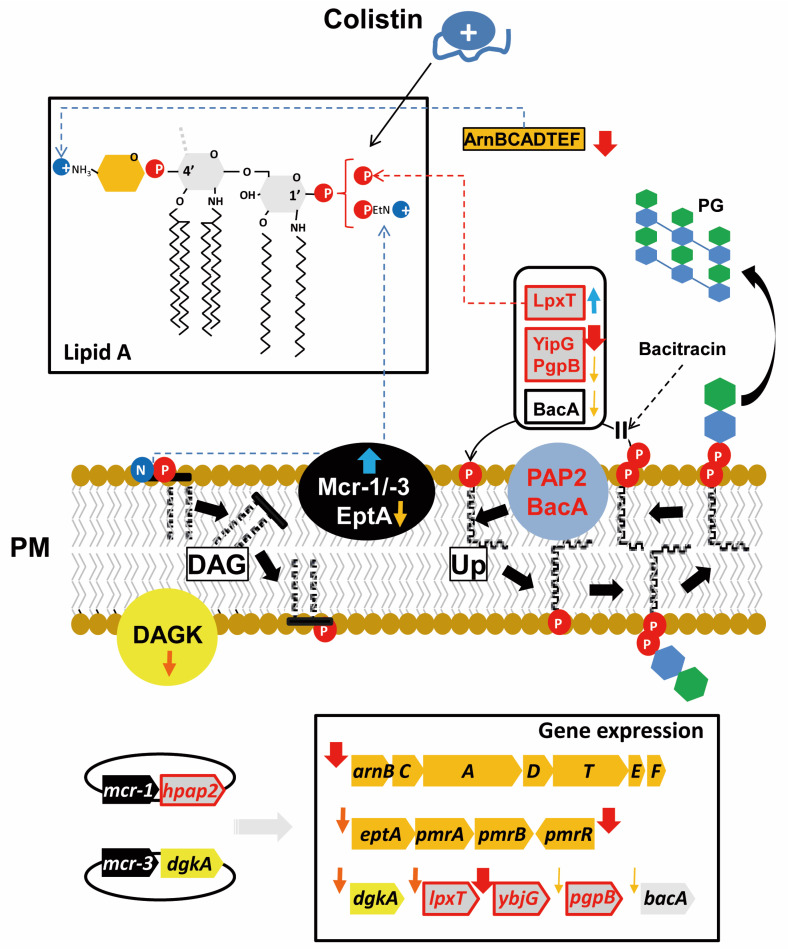
Landscape of phospholipid metabolism during expression of mcr-1/-3 genes. The plasmatic membrane (PM) is shown, with proteins involved in phosphoethanolamine (PEtN) transfer to lipid A and undecaprenyl phosphate (Up) and diacylglycerol (DAG) recycling positioned in their corresponding leaflets. Transcripts and (expected) protein regulations are shown by arrows, with color intensity and thickness indicating signaling strength: downward and red/orange, repression; upward and blue, induction. PG symbolizes peptidoglycan.

The strong effect of *mcr* gene expression on the regulation of phospholipid metabolism is, to our knowledge, shown in this work for the first time (Figure 5). Under this condition, the genes downregulated are *arnB, dgkA, eptA, pmrR, ybjG, bacA, lpxT*, and *pgpB*, with all but the last three being positively controlled by PmrAB (Figure 7) [2,3,6,7]. Since PEtN transferase activity would decrease LPS negative charge, *mcr* genes expressed in *E. coli* may interfere with iron signaling by reducing the negative charge of cellular surface, decreasing Fe^3+^ availability and the activation status of the PmrB sensor [3]. Thus, cells downregulated their endogenous systems for LPS modification with PEtN and L-Ara4N, tagged here by *arnB* and *eptA,* respectively (Figure 5A,B).

Between the two members of the *pap2* family also showing their mRNA accumulation to be decreased, *ybjG* and *lpxT*, the latter was unexpected since it was not previously found to be controlled by PmrAB [6]. However, the stronger repression of *pmrR* suggests that a significant amount of the LpxT enzyme could remain active to balance the lower expression of *ybjG* and, thus, the regulation mediated by PmrAB would adjust UPP recycling during LPS modification (Figure 7). Nevertheless, this condition might correspond to a disturbed metabolic status, since LpxT and MCR enzymes would compete for the same substrate, the 1′-phosphate lipid A, and that downregulation of the endogenous *dgkA* gene may limit DAG recycling.

**Figure 8:**
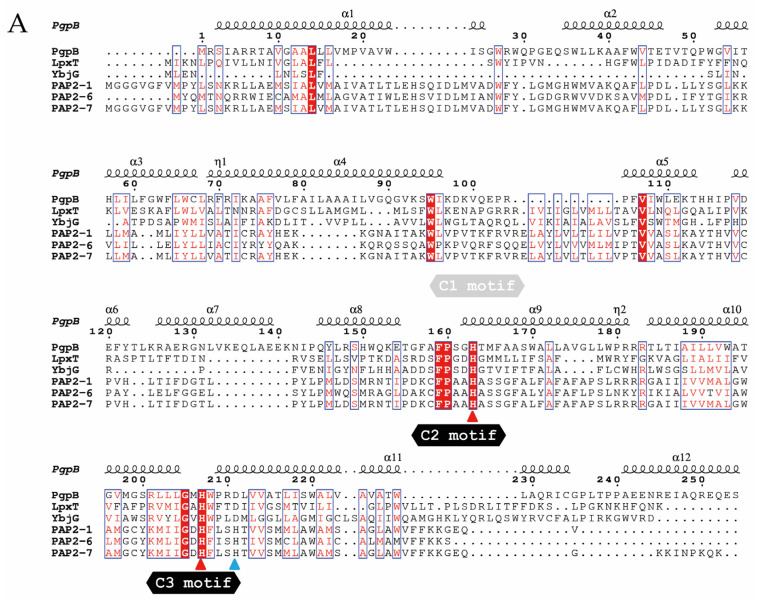

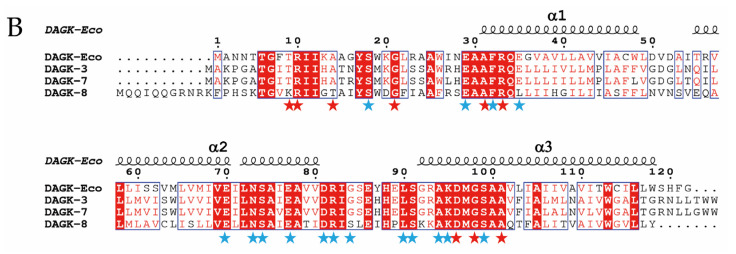
Structural determinants of hPAP2 and DAGK proteins. Multiple sequence alignments were performed by Clustal X 2.0 and emulated by EsPript 3.0. (**A**) Secondary structure elements of PgpB (4PX7) are shown above aligned PAP2 proteins, whose sequences are as follows: PgpB, NP_415362; LpxT, CQR81684; YbjG, WP_061361528; PAP2-1, WP_095885326; PAP2-6, OOS23616.1; PAP2-7, AUR80100. All sequences shown are from *E. coli* except for PAP2-6 (*Moraxella pluranimalium*) and PAP2-7 (*Klebsiella pneumoniae*). The three motifs involved in substrate binding are shown, with the catalytic triad His_C2_–Asp_C3_–His_C3_ indicated by red (conserved) or blue (non-conserved) triangles [7]. (**B**) Secondary structure elements of DAGK (2KDC) are shown above aligned proteins, whose sequences are as follows: DAGK-Eco, NP_418466.1; DAGK-3, WP_039026395.1; DAGK-7, AUR80099; DAGK-8, AVX52228. All sequences shown are from *E. coli* except for DAGK-7 and DAGK-8 (*K. pneumoniae*). Key residues for protein functioning that were previously determined are indicated by blue (role in activity) or red (role in folding) triangles [5].

Members of the PAP2 protein family, also known as lipid phosphate phosphatases (LPPs), are Mg^2+^-independent phosphatases that, in eukaryotes, hydrolyze phosphatidic acid to yield inorganic phosphate and diacylglycerol (DAG) for biosynthesis of triacylglycerol and phospholipids [18]. In prokaryotes, PAP2 proteins are functionally diverse and, among other functions, three enzymes from *E. coli* are involved in C_55_-P synthesis from UPP, a closely regulated committed step (Figure 7) [7]. C_55_-P is required as the biosynthetic platform for peptidoglycan synthesis, as well as for LPS modification by L-Ara4N, but Gram-negative bacteria can only synthetize it from UPP via its dephosphorylation. hPAP2 proteins encoded closely linked to *mcr-1-*like genes are far-related to UPP phosphatases from *E. coli* (Figure 8A). One of these, PgpB, is a phosphatidylglycerol phosphatase integrally located in the plasma membrane by six transmembrane helices, and its topological and 3D studies revealed a periplasm-oriented active site built by six residues from three conserved motifs C1, C2, and C3 [19,20,21]. These conform the phosphate binding and the catalytic triad signature, His_C2_–Asp_C3_–His_C3_, which is strictly conserved among the three PAP2 enzymes from *E. coli,* but not in hPAP2 proteins that mismatch the central residue of the motif (Figure 8A), suggesting different substrate specificities. Accordingly, the fact that *hpap2* expression in *E. coli* could not increase bacitracin resistance (Figure 6) may indicate that hPAP2 activity does not have UPP phosphatase activity unlike BacA, PgpB, or YbjG [21].

The moderated increase in bacitracin resistance conferred by the expression of *mcr-1* or *mcr-3* genes could be mediated by the strong decrease in *pmrR*, which may result in a higher level of LpxT activity, and/or by the reduction of the negative charge around bacterial cells that blocks interactions with positively charged antimicrobials. Thus, a hypothesis to contrast in the near future is that colistin and bacitracin co-resistance conferred by *mcr* genes may not be the result of co-selection but derived from the cross-talk between LPS decoration and UPP recycling.

Co-expression of *hpap2* and *mcr-1* genes is required for maximal expression of colistin resistance, although the increase is too modest to be detected in a classical, two-dilution fold, minimal inhibitory concentration (MIC) assay (Figure 4), which may explain why previous studies described contradictory results like a null effect or an essential role of *hpap2* expression in antimicrobial susceptibility [9,10]. hPAP2 belongs to the PAP2 family, which predicts membrane location, with active residues oriented toward the periplasm and a putative phosphatase activity on unknown substrate(s), which might not be UPP (see above) [7]. Clues about hPAP2 function might be derived from the homology to far-related sequences from *Francisella tularensis* and *Helicobacter pylori* [22,23,24]. Two PAP2 proteins, LpxE and LpxF, which are required for the natural resistance to colistin of these human pathogens, are phosphatases for 1′-phosphate or 4′-phosphate lipid A, respectively. Interestingly, LpxE from *H. pylori* is encoded closely linked and downstream the *eptA* ortholog from this organism (not shown), a genetic organization that invokes that of *mcr-1–hpap2*. Moreover, expression of LpxE or LpxF from *Francisella* in *E. coli* increases polymyxin resistance [22,23], although a similar role for hPAP2 could be ruled out since it does not produce a similar phenotype when *mcr-1* is not co-expressed (not shown). Since the strongest repression of *pmrR* was found after expressing *mcr-1*, and a relatively lower repression of *lpxT* was detected in cells carrying the *mcr-1–hpap2* construct, suggesting a higher availability of LpxT activity in this condition, the most consistent hypothesis for a role of hPAP2 in colistin resistance would be to convert 1′-bis-phosphate to 1′-phosphate lipid A, withdrawing the action of LpxT and increasing accessibility for PEtN addition by MCR-1-like enzymes (Figure 7).

In contrast to *hpap2* genes, the *dgkA* sequence closely linked to *mcr-3* determinant that was also shown to slightly improve colistin resistance (Figure 4) is closely related to its ortholog found in the genome of *E. coli* (Figure 8B). The hypothetical role of *dgkA* in colistin resistance can also be postulated on the basis of the strong conservation of their structural and functional determinants. Indeed, DAGK is the enzyme involved in recycling the membrane-disrupting DAG produced from PA by PEtN transferases like EtpA or MCR enzymes (Figure 7) [5]. *E. coli* DAGK is a protein integral in the plasma membrane with the active site oriented to the cytosol. All functional and structural determinants placed in the cytosol-contacting surface of its three helical segments are strongly conserved among the endogenous DAGK from *E. coli* and those encoded in the vicinities of *mcr-3*-like genes (Figure 7B), suggesting conservation of functionality. However, the *dgkA* gene linked to *mcr-8*, the less closely related genetic element, presents four polymorphisms in key residues for enzyme activity and/or folding of the protein [5].

This work presents evidence for the involvement of genes *hpap2* and *dgkA* in the function of *mcr-1* and *mcr-3*, two major determinants for colistin resistance. Their possible role may be to compensate for alterations of phospholipid metabolism during colistin resistance, which can provides clues to understand bacterial physiology and might contribute to a better control of antimicrobial resistance determinants.

## 4. Materials and Methods

### 4.1. Plasmid Constructs, Strains, and Growth Conditions

Specific primers (Table 1) were designed to amplify the full coding sequence of *mcr*-*1* (mcr1c), *mcr-1–hpap2* (mcr-pap2c), *mcr-3* (mcr3c), *mcr-3–dgkA* (mcr3dgkAc), and *hpap2* (pap2c). PCR was performed with High-Fidelity PCR Enzyme Mix (New England Biolabs, Ipswich MA, USA) according to the manufacturer’s protocol. PCR conditions for amplification included a primary denaturation step of 5 min at 98 °C, followed by 30 cycles of 30 s at 98 °C, 30 s at 59.5 °C for annealing, and 2 min at 72 °C for elongation, and a final elongation step of 10 min at 72 °C. DNA samples for amplification of PCR products were obtained by boiling overnight cultures. The *mcr-1* or *mcr-3* determinants were obtained from HSP38 or Eco46 strains, whose genomes were fully sequenced after their isolation from a human infection or from the feces of a healthy bovine, respectively [25,26]. PCR products were purified (MEGAquick-spin plus fragment DNA purification kit, iNtRON Biotechnology, Seongnam-Si Korea, Republic of (South)), digested with enzymes *Eco*RI and *Sal*I (New England Biolabs), and ligated (T4 DNA ligase, Thermofisher, Waltham MA, USA) to the arabinose-inducible pBAD24 vector (Life Science Market, Nova lifetech Limited, Hongkong). XL1 blue MRF’, a K12 derivative that was obtained from Statagene (Agilent, Santa Clara, CA, USA), was electroporated for selection of strains carrying recombinant plasmids according to classical methods [27]. XL1-Blue MRF’ cells carrying intact pBAD vector were used as a control strain for gene expression and antimicrobial resistance studies.

### 4.2. Quantitative PCR

Determination of the relative concentration of plasmids within cells was performed by real-time PCR using mcr1q or mcr3q primer pairs and recAq as a calibration reference (Table 1), whereas DNA samples were obtained using the non-selective boiling method (see above).

Gene expression analyses were performed with cells growing in liquid cultures (Mueller–Hinton broth supplemented with 100 mg/L ampicillin). After overnight growth, cultures were renewed by diluting 1/10 with fresh media supplemented with 0.2% arabinose and incubated at 37 °C with strong shaking (200 rpm). When cell cultures reached 0.3–0.5 optical density (OD) at 600 nm, they were quickly cooled on ice, centrifuged, and processed for RNA extraction (Aurum Total RNA Minikit, Bio-Rad), and then reverse-transcribed (PrimeScript™ RT reagent Kit, Takara); next, genomic DNA was removed (TURBO DNA-free kit, Ambion), according to the manufacturer’s protocols. SYBRgreen real-time quantitative assays were carried out using the SYBR^®^ Premix Ex Taq™ II (Tli RNase H Plus; Takara Bion Inc.) and an Applied Biosystems^®^ Step One PCR System. Oligo Primer Analysis Software v. 7 was utilized to design primer sequences with optimal amplification efficiencies (Table 1). The normalized relative quantities (NRQ) of transcripts were obtained using the 2^−ΔΔCt^ calculation method with the expression of *recA* gene used as a calibration reference, with every experimental condition including two technical replicates (duplicate reactions in the same qPCR) and three biological replicates from fully independent experiments. Ratios between the mean NRQ for every treatment (the different *mcr-1 ± hpap2* and *mcr-3 ± dgkA* constructs cloned in pBAD24 and transformed in XL1-Blue MRF’ cells) and control condition (XL1-Blue MRF’ cells carrying intact pBAD24 vector) and the standard error of the ratios were calculated according to previously reported methods [28].

### 4.3. Determination of Antibiotic Resistance

Colistin resistance was determined using cation-adjusted Mueller–Hinton agar (Sigma Aldrich). Liquid cultures (Mueller–Hinton broth) grown overnight with 100 mg/L ampicillin were renewed by diluting 1/10 with fresh media supplemented with 0.2% arabinose and incubated at 37 °C with strong shaking (200 rpm). When cell cultures reached OD_600 nm_ = 1.0, bacterial growth was diluted to 0.5 of the McFarland standard, corresponding approximately to OD_600 nm_ = 0.1 or 1.5 × 10^8^ CFU/mL (colony-forming units per mL), and 10-µL aliquots were spotted on Mueller–Hinton agar plates containing 0.02% arabinose and colistin or bacitracin at indicated concentrations, incubated at 37 °C overnight. The empty vector pBAD24 cloned in the same genetic background was used as a negative control. Colistin susceptibility was defined as the highest concentration of antibiotic that yielded visible growth of bacteria, whose values were confirmed using three independent experiments.

## 5. Conclusions

*hpap2* and *dgkA* genes closely linked to *mcr-1* and *mcr-3* might play a role on colistin resistance by compensating phospholipid metabolism functions altered during LPS modification by colistin resistance determinants.

## Figures and Tables

**Table 1:** Primer pairs and qPCR parameters.

Locus	Sequence 5′–3′ ^1^	Size ^2^	E ^3^	Corr. ^4^	Slope ^5^
mcr1c	F: AGTAGGAATTCATGATGCAGCATACTTCTG R: TAAGTCGACTGGAGTGTGCGGTGGGTTTGG	1685	-	-	-
mcr1pap2c	F: AGTAGGAATTCATGATGCAGCATACTTCTG R: AAAGTCGACTGAAAAAACCGTTCCGTAATA	2493	-	-	-
pap2c	F: CCATTGAATTCATGGGCGGTGGGGTGGG R: AAAGTCGACTGAAAAAACCGTTCCGTAATA	820	-	-	-
mcr3c	F: ATGGAGAATTCATGCCTTCCCTTATAAAAATA R: CCATGAAATACGTCGACAATAGTAATAAACCT	1714	-	-	-
mcr3dgkAc	F: ATGGAGAATTCATGCCTTCCCTTATAAAAATA R: AAAGGTCGACCTTATAATTAGCATCTATTGTT	2233	-	-	-
arnBq	F: ATTGGCAAGGGCGATGAA R: AGGCGTGACCATCAGCGTAT	126	102.55	0.997	−3.26
bacAq	F: CCGCGTGCGCCGGGTCTT R: ACCCGGCCACAGCGCCAG	84	103.5	0.999	−3.24
dgkAq	F: CTCAATAGCGCCATCGAAGC R: CGACGATAATGGCAATCAGCAC	112	100.64	0.997	−3.31
eptAq	F: ACGGCAACGGCAGTTT R: CCGCTCGCTGAATGATATCCA	112	95.1	0.996	−3.44
lpxTq	F: TGCTCTCTGTTCCCACGAAA R: ATAACGCCACATGAATGCC	95	99.32	0.998	−3.34
mcr1q	F: CATCGCTCAAAGTATCCAGT R: ACCATGTAGATAGACACCGTTC	115	97.307	0.999	−3.39
mcr3q	F: GACCGAGTACCTAACATCGAA R: CCTCGTCATAGCATGTGT	76	101.6	0.999	−3.28
pgpBq	F: GGCCGCGTCGGCGAACGT R: GCAGCAGGCGGCTTCCCA	75	101.0	0.998	−3.30
pmrRq	F: ATGAAAAACCGTGTTTATGA R: TCAGTACGTGGCAAACCA	90	103.9	0.999	−3.23
recAq	F: GTATGATGAGCCAGGCGATGC R: GCGCGTTACCACCGGTAGTG	138	100.38	1.0	−3.31
ybjGq	F: ACGCGACGCCAGACTCGG R: CACGGCCAGCAACGGCAC	89	101.2	0.999	−3.30

^1^ Underlined sequences indicate restrictions sites used for cloning in pBAD24 (*Eco*RI, *Sal*I). ^2^ Amplicon lengths (bp). ^3^ Efficiency of the qPCR. ^4^ Correlation (*R*^2^) of the qPCR calibration. ^5^ Calibration of the qPCR: slope.
